# Supplementary material for: Inflammatory factors and risk of lung adenocarcinoma: a Mendelian randomization study mediated by blood metabolites
Source: Front Endocrinol (Lausanne). 2024 Aug 27;15:1446863. doi: 10.3389/fendo.2024.1446863 (PMC11384989; doi:10.3389/fendo.2024.1446863)
Supplement: Supplementary file 1 [file DataSheet1.zip › supplementary files/S2/GCST90200192/forest.pdf]

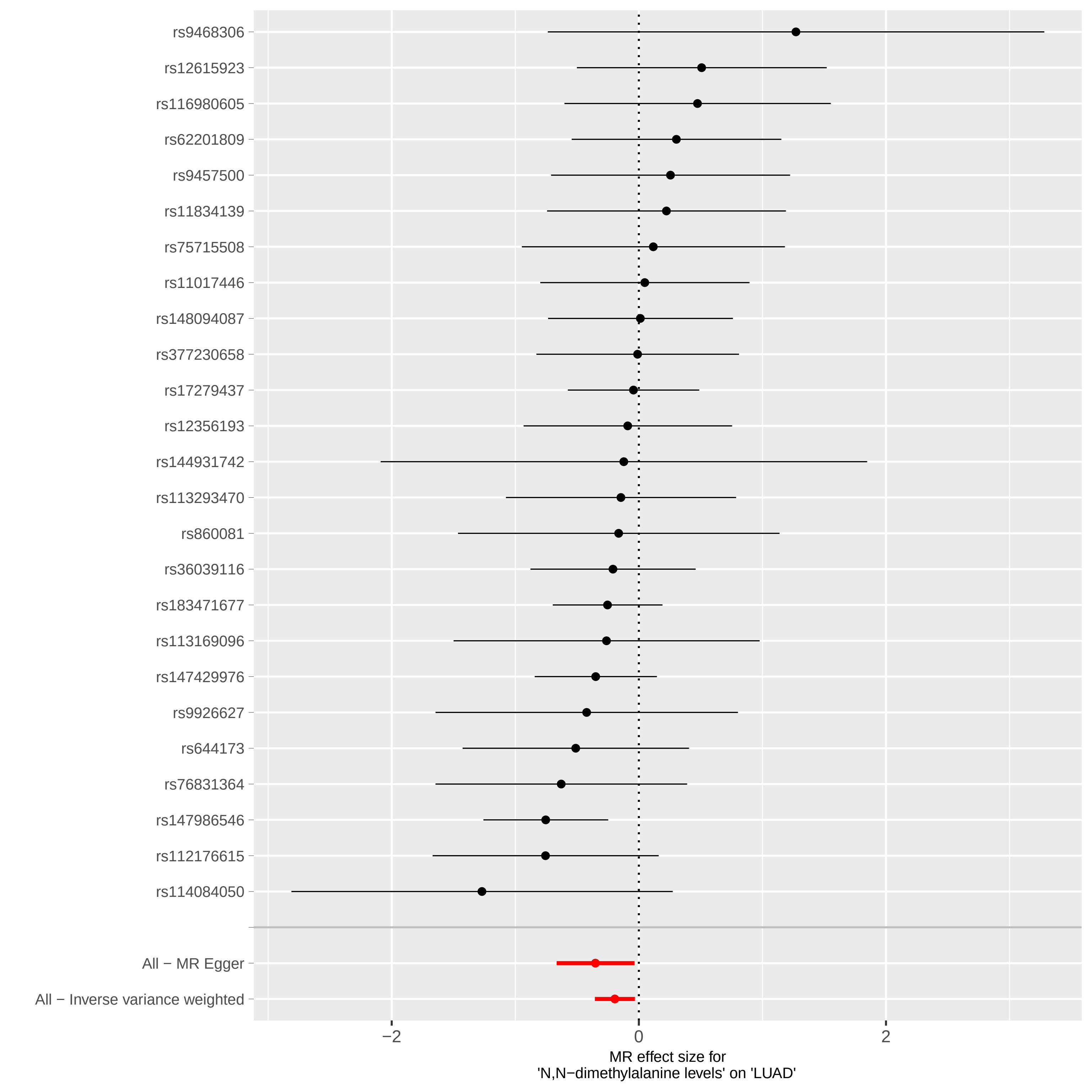
rs9468306
rs12615923
rs116980605
rs62201809
rs9457500
rs11834139
rs75715508
rs11017446
rs148094087
rs377230658
rs17279437
rs12356193
rs144931742
rs113293470
rs860081
rs36039116
rs183471677
rs113169096
rs147429976
rs9926627
rs644173
rs76831364
rs147986546
rs112176615
rs114084050
All − MR Egger
All − Inverse variance weighted
−2
0
2
MR effect size for
'N,N−dimethylalanine levels' on 'LUAD'
